# Inflammatory Bowel Disease and Mental Health: The Complex Relationship Between Gut and Brain

**DOI:** 10.7759/cureus.98357

**Published:** 2025-12-03

**Authors:** Talal Zahid, Sohail Wahid, Salman Idrees Bhutta, Mohamed Bashir, Vikram Kumar, Mohamed Abdelfattah, Omar Saafan, Ali Javeed, Abdul Hadi Hasan, Neil Russel Dsouza, Fatima Ali

**Affiliations:** 1 Internal Medicine, Faisalabad Medical University, Faisalabad, PAK; 2 Internal Medicine, Cork University Hospital, Cork, IRL; 3 Gastroenterology, Gomal Medical College, Dera Ismail Khan, PAK; 4 Gastroenterology, Aberdeen Royal Infirmary Hospital NHS, Aberdeen, GBR; 5 Internal Medicine, Scarborough General Hospital, Scarborough, GBR; 6 Internal Medicine, Liaquat University of Medical and Health Sciences, Jamshoro, PAK; 7 Internal Medicine, York and Scarborough Teaching Hospitals NHS Foundation Trust, Scarborough, GBR; 8 Internal Medicine, York and Scarborough Teaching Hospitals HNS Foundation Trust, Scarborough, GBR; 9 General Medicine, Scarborough General Hospital, Scarborough, GBR

**Keywords:** anxiety, depression, gut heath, inflammatory bowel disease, quality of life

## Abstract

Background: The study sought to determine the association between IBD severity and mental health impact as measured by anxiety, depression, and QOL in our Pakistani patients.

Design and Methods: A cross-sectional study was conducted with 400 IBD patients from clinics and community healthcare centres in Faisalabad, Dera Ismail Khan, and Karachi. Data were collected using the Short IBD Questionnaire (SIBDQ) and Hospital Anxiety and Depression Scale (HADS). Spearman correlations, chi-square tests, Mann-Whitney U, Kruskal-Wallis, and multiple regression analyses (SPSS v26) were performed.

Results: Of 400 participants, 240 (60%) were female, and most were aged 25-34 years (126, 31%). A total of 190 (47%) scored ≥11 on the HADS, indicating clinically significant anxiety or depression. SIBDQ and HADS scores were negatively correlated (ρ = −0.33, 95% CI −0.40 to −0.26, p < 0.001). Multiple regression showed that higher HADS scores were predicted by greater disease severity (B = 0.19, 95% CI 0.08-0.29, p = 0.001), female gender (B = 0.89, 95% CI 0.25-1.53, p = 0.007), younger age (B = −0.21, 95% CI −0.37 to −0.05, p = 0.011), disease activity (B = 0.44, 95% CI 0.14-0.74, p = 0.004), IBD type (B = 0.53, 95% CI 0.16-0.90, p = 0.006), and family history (B = 0.69, 95% CI 0.08-1.30, p = 0.026). Patients with severe IBD had the highest rates of clinically significant anxiety or depression (62% vs 43% in moderate and 10% in normal; χ²(4) = 81.9, p < 0.001). Females and younger participants reported greater disease severity and mental health burden (p < 0.01).

Conclusions: Anxiety and depression are prevalent among Pakistani IBD patients, particularly in women, younger adults, and those with severe disease. These findings highlight the need for integrated gastroenterology-psychiatry care with routine mental health screening and psychosocial support to improve long-term outcomes and quality of life.

## Introduction

Inflammatory bowel diseases (IBD) are chronic conditions characterised by inflammation of the gut of unknown cause. They are currently mainly classified into ulcerative colitis (UC), Crohn's disease (CD), and an intermediate type [1]. IBD pathophysiology is characterised by imbalances in innate and adaptive immune responses, driven by genetic predisposition, epigenetic, and environmental factors [2]. There are two prevailing hypotheses to explain the pathogenesis of IBD: immune dysregulation leading to abnormal responses to normal microflora and an altered gut microbiota or defects in the epithelial barrier that result in aberrant immune activation [3].

IBD is now a global disease, and the prevalence exceeds 0.3% in many industrialised countries. Although the incidence is declining in the Western world, it is increasing in newly industrialised areas, underscoring the growing global burden and the urgency of developing more effective preventive and treatment modalities [4]. Globally, IBD incidence is rising. According to GBD 2021 data, age-standardised incidence continues to climb in Asian regions, including South Asia, highlighting a growing regional burden [5]. Modelling studies predict that the number of prevalent cases in South Asia (India) will rise from ~212,000 in 2017 to ~2.2 million by 2035 [6]. In the South Asian region, systematic review data confirm that IBD is an emerging disease, with a higher Ratio of UC to CD and modest familial aggregation of cases [7]. However, epidemiological data from Pakistan remain limited, highlighting the need for local studies to assess disease burden and mental health impact.

Psychiatric comorbidities, such as depression, anxiety, bipolar disorder, substance use, and suicidal risk, are frequently coexistent in IBD patients, with burden varying according to disease activity and extent [8,9]. These comorbidities are associated with higher hospitalisations, emergency visits, and healthcare costs. Early detection and coordinated care could lead to better patient outcomes at lower cost [10]. In many studies, anxiety and depression are found in approximately 20-30% of IBD patients, and they exert a significant negative impact on health-related quality of life (HRQoL), independent of disease activity [11,12].

In this investigation, we evaluate the complex relationship between IBD and mental health, with a focus on gut-brain communication mechanisms, to develop interventions that improve patient outcomes and provide culturally appropriate care, emphasising the integration of physical and psychological management.

Rationale

IBD, such as Crohn's disease and ulcerative colitis, is a progressive gastrointestinal (GI) disorder whose prevalence is increasing in South Asia, including Pakistan, potentially due to urbanisation, dietary changes, and environmental factors. According to GBD 2021 data, age-standardised incidence continues to rise in Asian regions, including South Asia [5], and projection studies predict prevalent cases in India will increase from ~212,000 in 2017 to ~2.2 million by 2035 [6]. Aside from its physical burden, IBD is closely associated with mental health conditions such as anxiety, depression, and stress, which can exacerbate disease activity and impair quality of life. In Pakistan, these psychological effects are less recognised due to social stigma, limited mental health services, and cultural constraints that hinder care-seeking. Therefore, this study aims to quantify the prevalence of anxiety and depression in relation to IBD severity in a Pakistani population, to inform integrated care approaches addressing both physical and psychological health.

Study objectives

The main aim of this research is to examine the relationship between inflammatory bowel disease (IBD) and comorbid mental illnesses, particularly anxiety and depression, in Pakistani patients. Specifically, the study aims to:

(1) Assess the prevalence of anxiety and depression among patients with IBD; (2) examine the association between IBD severity and psychological burden, as measured by HADS; (3) determine the association of demographic and clinical factors-including age, gender, IBD type, and disease duration-with HADS scores.

## Materials and methods

Methodology

A cross-sectional study was carried out to investigate the association between IBD and mental health outcomes, anxiety, and depression among Pakistani patients. The study was approved by the Institutional Review Board of Faisalabad Medical University (062s-IRB-FMU-2025). The information was obtained from gastroenterology clinics, outpatient departments, and community-based health facilities in Faisalabad, Dera Ismail Khan, and Karachi. These sites predominantly attract demographically diverse populations of adults (including those across the adult lifespan and both genders) with a wide range of socioeconomic, educational, and background profiles. A convenience sampling strategy was employed, which may have overrepresented women, younger adults, and urban-dwelling patients, potentially limiting generalizability to rural or older populations. 

Sample size and technique

The target population was assumed to be infinite, as the precise number of adults with IBD in Pakistan was unknown. The sample size was calculated using the standard formula for estimating a proportion:

\[n = \frac{Z^2 \cdot p (1 - p)}{d^2}\]

Where Z is the z-score corresponding to a 95% confidence level (1.96), ppp is the estimated prevalence (0.5, chosen for maximum sample size in the absence of reliable local data), and ddd is the acceptable margin of error (0.05) [13]. This yielded a minimum sample size of 384; 400 participants were recruited to account for potential non-response.

We used a convenience sampling approach to enrol participants from gastroenterology clinics, outpatient departments, and community health centres in Faisalabad, Dera Ismail Khan, and Karachi. Data collectors approached eligible patients, explained the study's purpose, and obtained consent before enrollment. Participants did not receive payment or any material reward for participating.

Participant eligibility criteria

The participants in the study were patients aged 18 years and over with a confirmed diagnosis of IBD (Crohn's disease or UC) based on a gastroenterologist's review of their medical records, and who were being treated or followed up at the participating clinics or hospitals. Additionally, participants had to be capable and willing to provide informed consent. Participants were excluded if they had severe cognitive impairment, a primary psychiatric diagnosis (e.g., schizophrenia, bipolar disorder) that would interfere with questionnaire completion, significant non-IBD gastrointestinal conditions, severe acute illness, inpatient status, or were pregnant or breastfeeding.

Research tools and measures

A structured questionnaire was used for data collection, comprising three parts: sociodemographic characteristics and a mental health assessment. The data were collected using a demographic information form prepared by the researchers, the Short Inflammatory Bowel Disease Questionnaire (SIBDQ), and the Hospital Anxiety and Depression Scale (HADS). Although these scales were not formally translated into Urdu, research staff provided bilingual assistance and clarified items verbally as needed to ensure participants’ comprehension. Future studies should consider translating and culturally validating these measures for broader use in Pakistani populations.

Demographic information

The first section included demographic and clinical data, such as age, sex, marital status (single or married), education level (less than university/beyond university), occupation, IBD type, duration (in years), comorbidities, and treatment modalities. These were potentially relevant in explaining the causes of psychological symptoms and QOL and allowed for subgroup analyses (see Appendix 1).

Participants self-reported their current IBD activity over the preceding four weeks, categorizing their disease as mild, moderate, or severe based on their own perception of symptom severity (e.g., frequency of abdominal pain, diarrhea, or blood in stool). No objective clinical, endoscopic, or laboratory assessments were performed.

Short Inflammatory Bowel Disease Questionnaire (SIBDQ)

The SIBDQ, developed by Irvine et al. in 1996, is a 10-item questionnaire that measures health-related quality of life (HRQol) in patients with inflammatory bowel disease (IBD). The questionnaire consists of four domains: bowel symptoms, systemic symptoms, emotional function, and social function. A higher score, indicating a better QOL, is obtained for each item using a 7-point Likert scale. The total score ranges from 10 to 70. For this study, a score of ≤50 was considered indicative of lower HRQoL, while scores >50 indicated higher HRQoL. It exhibits good internal consistency, with a Cronbach's alpha coefficient typically greater than 0.78 in many populations [14] (see Appendix 2). Permission to use the Inflammatory Bowel Disease Questionnaire (IBDQ) was obtained from McMaster University under an official license agreement (License Ref: IBDQ26-038).

Hospital Anxiety and Depression Scale (HADS)

Anxiety and depressive symptoms of IBD patients were evaluated using HADS. The scale is a 14-item scale with equal items about anxiety and depression, rated on a four-point Likert scale (0-3); higher scores reflect more severe symptoms. Developed by Zigmond and Snaith (1983), the HADS has been shown to have good reliability (α = 0.83 for anxiety; α = 0.82 for depression, Cronbach's α) [15] (see Appendix 3). The Hospital Anxiety and Depression Scale (HADS) was used for the assessment of anxiety and depression. Official permission and user license for the scale were obtained from Mapi Research Trust (Work Order No. 2511718).

Procedure

Over six months (March-August 2025), all patients attending outpatient services at public hospitals, gastroenterology clinics, and community health centres in Faisalabad, Dera Ismail Khan, and Karachi were recruited. Patients meeting the inclusion criteria were presented with information about the study, and written consent was obtained. Participants independently or with the assistance of trained research staff filled out a self-report questionnaire that included a demographic form, the HADS, and the SIBDQ. Anonymity was maintained, and privacy/confidentiality were respected as appropriate in terms of locale-specific ethical/cultural traditions. 

Participants completed the questionnaires independently or with the assistance of trained research staff, who received standardised instructions on administering the measures. Missing items were minimal (<5%) and handled using mean imputation for scales if ≤2 items were missing; otherwise, the questionnaire was excluded from analysis.

Data analysis

The data analysis was conducted using IBM SPSS Statistics version 26 (IBM Corp., Armonk, NY). To describe both the demographic and clinical aspects of the participants, frequencies and percentages were determined. Q-Q plots were used to determine the trend in SIBDQ and HADS scores by detrending them. The Spearman rank-order correlation was used to determine the correlation between SIBDQ and HADS scores. Gender differences in SIBDQ and HADS were analysed using the Mann-Whitney U test, whereas the Kruskal-Wallis test was employed to determine differences among the age groups. To determine the predictors of mental health burden (HADS scores) based on SIBDQ, age, sex, type of IBD, current disease activity, and family history of IBD, multiple linear regression analysis was used. For this study, “family history of IBD” was defined as having at least one first-degree relative (parent, sibling, or child) with a medically confirmed diagnosis of IBD. The chi-square test of independence was employed to assess the relationship between IBD severity and HADS categories, and Cramér's V was used as a measure of effect size. Statistical tests were all two-tailed, and a p-value of less than 0.05 was regarded as significant.

Ethical considerations

All individuals were briefed on the study's objectives, procedures, potential hazards, and benefits in a language they could understand; written informed consent was obtained before their participation. The study was conducted on a voluntary basis, and participants were free to withdraw at any time without affecting their future medical treatment. No identification or individual participant data were collected, and all data were coded during entry, processing, and analysis to maintain confidentiality. The dignity, privacy, and welfare of all those involved were preserved throughout the entire period.

## Results

Table 1 has the demographic and clinical features of the 400 respondents. Among 400 participants with IBD, most were 18-34 years old (n=216, 54%) and female (n=240, 60%). Over half were unmarried (n=220, 55%) and had diverse educational backgrounds. IBD types included ulcerative colitis (n=178, 44%), Crohn’s disease (n=48, 12%), and indeterminate (n=174, 43%), with 262 (66%) reporting moderate to severe disease activity. Treatments included 5-ASA (n=164, 41%), biologics (n=82, 20%), immunomodulators (n=44, 11%), and steroids (n=32, 8%); 78 (20%) received no treatment. About half had a history of IBD-related surgery (n=205, 51%) or a family history (n=218, 55%). Psychological burden was notable: 374 (94%) had received some psychiatric care, and HADS scores indicated 190 (47%) as cases, 130 (33%) borderline, and 80 (20%) normal. SIBDQ scores classified 210 (52%) as severe, 140 (35%) moderate, and 50 (13%) normal, reflecting substantial impact on quality of life.

**Table 1:** Participant Demographics (N = 400) Values represent frequency (n) and percentage (%) of participants (N = 400)

Variable	f (N)	%
Age	-	-
18-24 years	90	22
25-34 years	126	31
35-44 years	67	17
45-54 years	27	7
55-64 years	35	9
≥65 years	55	14
Gender	-	-
Male	160	40
Female	240	60
Marital status	-	-
Single	220	55
Married	66	17
Divorced/widowed	114	28
Educational level	-	-
No formal education	124	31
Primary	60	15
Secondary/intermediate	85	21
Bachelor's	93	23
Postgraduate	38	9
Employment	-	-
Student	48	12
Employed	146	36
Homemaker	79	20
Unemployed/ retired	127	32
Type of inflammatory bowel disease	-	-
Ulcerative colitis	178	44
Crohn's disease	48	12
Indeterminate	174	43
Duration since diagnosis	-	-
<1 year	137	34
1-3 years	69	17
4-6 years	70	18
≥7 years	124	31
Current disease activity (self-reported)	-	-
In remission / no symptoms	81	20
Mild	57	14
Moderate	179	45
Severe	83	21
Current treatment	-	-
5-ASA (mesalazine)	164	41
Steroids	32	8
Immuno-modulators	44	11
Biologics	82	20
No treatment	78	20
History of inflammatory bowel disease-related surgery	-	-
Yes	205	51
No	195	49
Family history of inflammatory bowel disease	-	-
Yes	218	55
No	182	45
Any psychiatric treatment in past or present?	-	-
No	26	6
Yes, counselling/therapy	138	35
Yes, medication	153	38
Both	83	21
Inflammatory bowel disease severity	-	-
Severe (≤45)	210	52
Moderate (46–59)	140	35
Normal (≥60)	50	13
Hospital Anxiety and Depression Scale categories	-	-
Normal (0–7)	80	20
Borderline (8–10)	130	33
Case (≥11)	190	47

**Figure 1:**
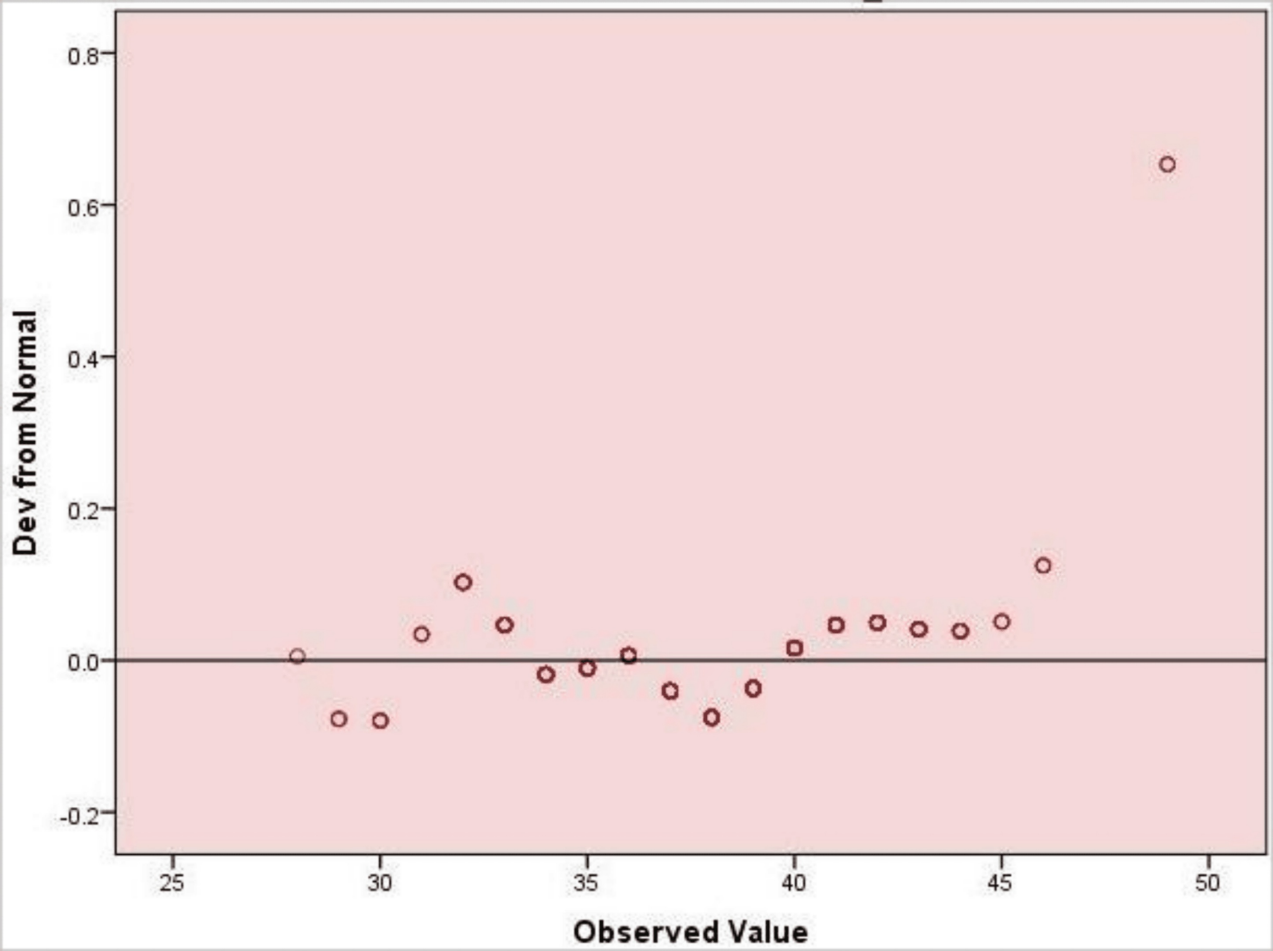
Detrended Q–Q Plot of Total SIBDQ Scores (N = 400) The plot illustrates deviations of observed values from a normal distribution. Data points closely aligned with the horizontal reference line indicate approximate normality, while deviations reflect departures from the normal distribution. SIBDQ: Short Inflammatory Bowel Disease Questionnaire

Figure 1 shows that SIBDQ scores were approximately normally distributed, with minor deviations at the upper end. This supports the use of nonparametric tests where appropriate.

**Figure 2:**
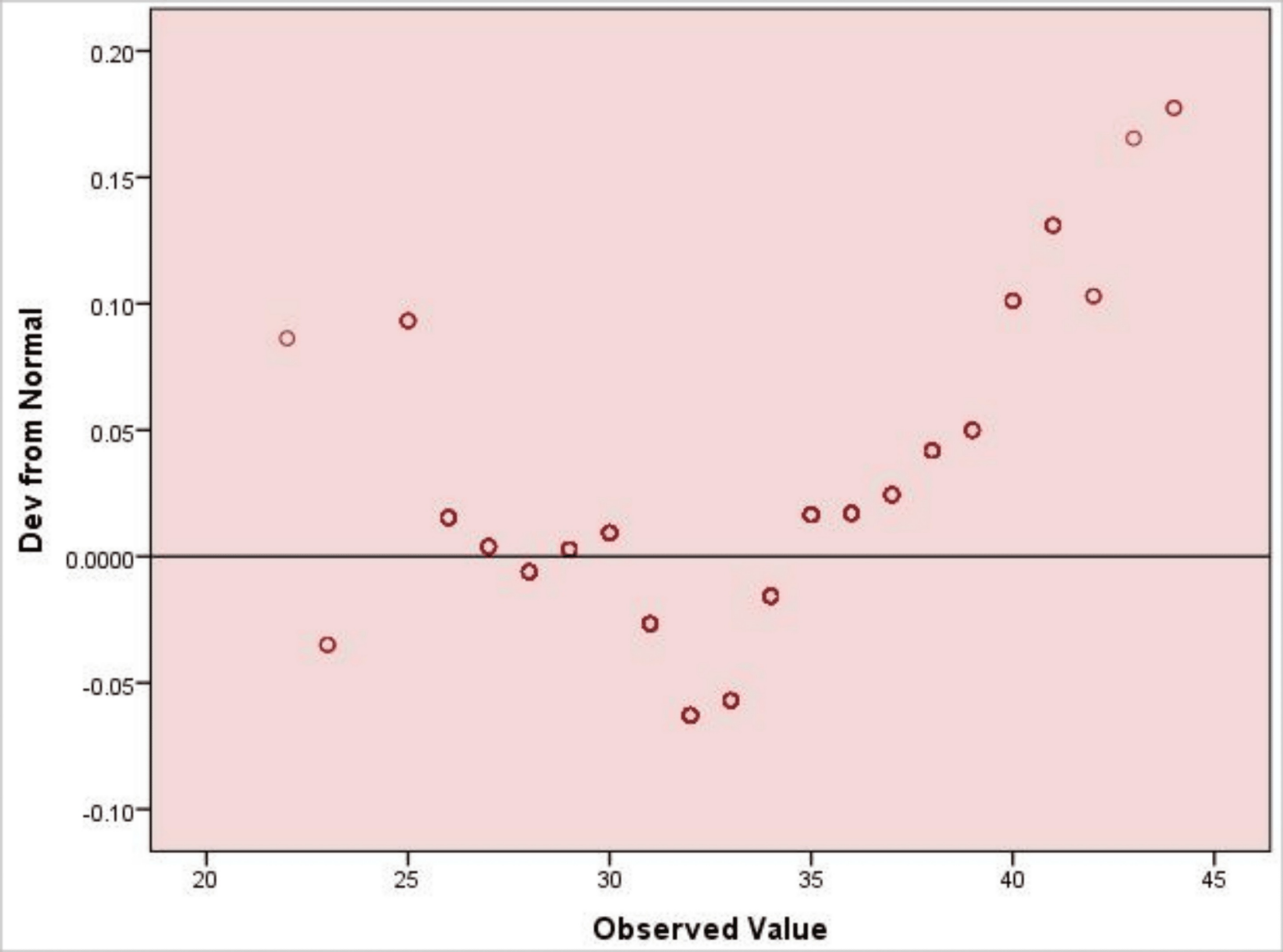
Detrended Q–Q Plot of HADS Scores (N = 400) The figure displays deviations of observed HADS total scores from a normal distribution. Points close to the horizontal reference line suggest approximate normality, whereas systematic deviations indicate departures from normality. HADS: Hospital Anxiety and Depression Scale

Figure 2 indicates that HADS scores were not normally distributed, supporting the choice of nonparametric tests for subsequent analyses.

Table 2 presents the Spearman correlation between SIBDQ and HADS scores (N = 400). There was a significant negative correlation (ρ = −0.33, 95% CI −0.40 to −0.26, p < 0.001), showing that lower SIBDQ scores were associated with higher HADS scores. 

**Table 2:** Spearman's Correlations Among Study Variables (N = 400) Values are Spearman's rank-order correlation coefficients (ρ); p <0.001 (**), N = 400; all percentages used in descriptive analysis are based on valid responses (i.e., excluding missing data) to ensure consistency across variables. SIBDQ: Short Inflammatory Bowel Disease Questionnaire; HADS: Hospital Anxiety and Depression Scale

Variables	ρ	t(df)	p
SIBDQ	-	-	-
HADS	-0.33	-6.87 (398)	< 0.001^**^

Table 3 shows gender differences in IBD severity (SIBDQ) and mental health burden (HADS) among 400 participants. Females (N = 240, 60%) had significantly lower SIBDQ scores than males (median (IQR): 52 (46-58) vs. 57 (52-63); U = 15,960, Z = -2.54, p = 0.011), indicating poorer quality of life. They also had higher HADS scores (median (IQR): 19 (15-24) vs. 16 (12-20); U = 23,184, Z = -3.12, p = 0.002), reflecting greater anxiety and depression. These findings indicate that female participants reported lower SIBDQ scores and higher HADS scores than males, suggesting an association between gender and both disease-specific quality of life and psychological burden.

**Table 3:** Gender Differences in IBD Severity (SIBDQ) and Mental Health Burden (HADS) Based on Mann–Whitney U Tests (N = 400) Note: N = 400 (Males = 160, 40%; Females = 240, 60%); Mann–Whitney U test was used for all comparisons; p-values marked with *, ** indicate statistical significance at p <0.05*, <0.01**. SIBDQ: Short Inflammatory Bowel Disease Questionnaire; HADS: Hospital Anxiety and Depression Scale; IBD: inflammatory bowel disease

Variable	N	Mean Rank	Median (IQR)	Sum of Ranks	Mann-Whitney U	Wilcoxon W	Z	p
SIBDQ								
Male	160	220.75	57 (52–63)	35,320	22,440	15,960	–2.54	0.011*
Female	240	187	52 (46–58)	44,880	15,960	44,880	–2.54	0.011*
HADS								
Male	160	175.6	16 (12–20)	28,096	15,216	23,184	–3.12	0.002**
Female	240	217.1	19 (15–24)	52,104	23,184	52,104	–3.12	0.002**

Table 4 shows age-related differences were observed in IBD severity (SIBDQ) and psychological burden (HADS). Younger participants (18-24 years, N = 90; 25-34 years, N = 126) had higher SIBDQ (median 60 (IQR 55-65) and 58 (53-63)) but also higher HADS scores (median 22 (18-26) and 20 (16-24)), indicating better quality of life yet greater anxiety/depression. The ≥65 years group (N = 55) had the lowest SIBDQ (43 (37-49)) and HADS (12 (8-16)), reflecting relatively lower HRQoL but less psychological distress. Differences were significant for both SIBDQ (χ²(5) = 117.1, p < 0.001) and HADS (χ²(5) = 128.3, p < 0.001).

**Table 4:** Age Group Differences in IBD Severity (SIBDQ) and Mental Health Burden (HADS) Based on Kruskal–Wallis Tests (N = 400) N = number of participants in each age category; % = percentage of the total sample; percentages are based on total N = 400; values are mean ranks from Kruskal–Wallis H tests. Overall test statistics are reported in the bottom row for each dependent variable: total SIBDQ (χ²(5) = 117.1, <0.001**) and total HADS (χ²(5) = 128.3, <0.001**); significance levels: p <0.001** HADS: Hospital Anxiety and Depression Scale; SIBDQ: Short Inflammatory Bowel Disease Questionnaire; IBD: inflammatory bowel disease

Variables	N	Mean Rank	Median (IQR)	χ² (df = 5)	p
Total SIBDQ				117.1	<0.001**
18-24 years	90	235.4	60 (55-65)		-
25-34 years	126	225.75	58 (53-63)	–	-
35-44 years	67	220.3	56 (51-61)	-	-
45-54 years	27	170.85	48 (42-54)	-	-
55-64 years	35	165.2	46 (40-52)	-	-
≥65 years	55	150.4	43 (37-49)	-	-
Total HADS				128.3	<0.001**
18-24 years	90	260.4	22 (18-26)	-	-
25-34 years	126	240.1	20 (16-24)	-	-
35-44 years	67	215.75	18 (14-22)	-	-
45-54 years	27	185.25	16 (12-20)	-	-
55-64 years	35	160.1	14 (10-18)	-	-
≥65 years	55	145.85	12 (8-16)	-	-

Table 5 presents the multiple regression predicting mental health burden (HADS) from demographic and clinical variables in 400 participants. Higher HADS scores were significantly associated with greater IBD severity (SIBDQ; B = 0.19, β = 0.16, p = 0.001), younger age (B = -0.21, β = -0.11, p = 0.011), female gender (B = 0.89, β = 0.14, p = 0.007), IBD type (B = 0.53, β = 0.13, p = 0.006), current disease activity (B = 0.44, β = 0.14, p = 0.004), and positive family history (B = 0.69, β = 0.09, p = 0.026). These results indicate that both clinical factors (severity, type, activity, family history) and demographic factors (age, gender) were independently associated with mental health burden in patients with IBD.

**Table 5:** Multiple Regression Predicting Mental Health Burden (HADS) From Demographic and Clinical Variables (N = 400) Note. N=400, B = unstandardized regression coefficient; SE = standard error; β = standardised regression coefficient; CI = confidence interval; LL = lower limit; UL = upper limit; IBD = inflammatory bowel disease; All predictors were entered; p<0.05*, p <0.01**. HADS: Hospital Anxiety and Depression Scale; SIBDQ: Short Inflammatory Bowel Disease Questionnaire; IBD: inflammatory bowel disease

Predictor	B	SE	β	t	p	95% CI LL	95% CI UL
Constant (Total HADS)	20.51	2.21	—	9.28	<0.001^**^	16.17	24.85
Total SIBDQ	0.19	0.06	0.16	3.17	0.001^**^	0.08	0.29
Age	–0.21	0.08	–0.11	–2.56	0.011^*^	–0.37	–0.05
Gender (ref = male)	0.89	0.33	0.14	2.72	0.007^**^	0.25	1.53
IBD type	0.53	0.19	0.13	2.79	0.006^**^	0.16	0.90
Current disease activity (self-reported)	0.44	0.15	0.14	2.93	0.004^**^	0.14	0.74
Family history of IBD	0.69	0.31	0.09	2.23	0.026^*^	0.08	1.30

Table 6 presents the cross-tabulation of IBD severity and HADS categories in 400 participants. There was a significant association between IBD severity and mental health burden (χ²(4) = 81.9, Cramer's V = 0.32, p < 0.001). Among participants with severe IBD (N = 210, 52%), the majority were HADS cases (130, 62%), whereas those with moderate IBD (N = 140, 35%) had a more even distribution across HADS categories. Participants with normal IBD severity (N = 50, 12%) were mainly in the normal HADS category (30, 60%). These findings indicate that higher IBD severity is strongly associated with increased anxiety and depression.

**Table 6:** Cross-tabulation of IBD Severity and HADS Categories (N = 400) Values are presented as N (%); Totals represent row sums; χ² (4) = 81.9, p = <0.001**, indicating a statistically significant association between Inflammatory Bowel Disease Severity and Hospital Anxiety and Depression Scale Categories, N = 400. HADS: Hospital Anxiety and Depression Scale; IBD: inflammatory bowel disease

IBD Severity	HADS Normal (0–7)	HADS Borderline (8–10)	HADS Case (≥11)	Total	x^2^ (4)	Cramer's V	p
Severe (≤45)	20 (9%)	60 (29%)	130 (62%)	210 (52%)	-	-	-
Moderate (46–59)	25 (18%)	55 (39%)	60 (43%)	140 (35%)	-	-	-
Normal (≥60)	30 (60%)	15 (30%)	5 (10%)	50 (12%)	-	-	-
Total	75 (19%)	130 (32%)	195 (49%)	400 (100%)	81.9	0.32	<0.001^**^

## Discussion

The present study investigated the correlation between IBD and the burden of mental health among Pakistani patients with anxiety, depression, and QOL as the main variables. We found that there was a moderate negative relationship between HADS and SIBDQ, which suggested that the more a person had anxiety and depression, the worse their quality of life was. These results were also consistent with a prospective cohort study that found a significant negative correlation between HADS scores and SIBDQ scores among IBD patients [16].

Women in our study reported a significantly higher HADS score, which indicated greater psychological distress and a poorer quality of life based on the lower SIBDQ score than males. An earlier study supported these results as well, as it also identified higher anxiety and depression rates, low quality of life, and slightly poorer sleep quality among female IBD patients compared to males [17].

Age-related patterns were observed in both IBD severity and psychological burden. Younger participants (18-34 years) had higher SIBDQ scores, reflecting better disease-specific quality of life, but also reported higher HADS scores, indicating higher levels of anxiety and depression. Older participants, particularly those ≥65 years, had lower SIBDQ scores than younger adults, suggesting relatively lower HRQoL, as well as lower HADS scores. These results indicate that age was associated with both HRQoL and psychological burden, with younger adults reporting greater psychological burden despite higher perceived quality of life. This pattern aligns with prior literature suggesting differences in coping strategies and emotional regulation across age groups [18,19].

Our findings further indicate that both clinical and demographic factors independently contribute to mental health burden. Active disease, IBD type, and family history were associated with higher HADS scores, consistent with literature showing that disease activity and psychosocial stressors strongly influence anxiety and depression, potentially via heightened stress responses, maladaptive coping, or alterations in the microbiota-gut-brain axis [20-22]. Female gender and younger age were also predictive of higher psychological burden, suggesting that interventions targeting these groups may be particularly beneficial. The observed associations underscore the complex biopsychosocial interplay in IBD, where disease severity, genetic predisposition, and psychosocial environment collectively impact mental health. We also found that the severity of IBD is related to the severity of anxiety and depression, as previous studies found that patients with more active disease or poorer quality of life have higher levels of psychological distress even during remission periods [23]. This highlights the strong association between physical disease activity and the mental health of IBD patients.

The implications of these findings on Pakistani clinical practice are that psychological screening and combined mental health support should be a routine in IBD care, especially in women and younger patients at higher risk. The observed psychological burden could be reduced through culturally tailored interventions, patient education, and conveniently accessible mental health care, thereby enhancing HRQoL. Nevertheless, several limitations should be taken into account when interpreting these findings. The cross-sectional design does not allow causal inference, and convenience sampling of urban centres does not allow generalisation to rural populations or patients with limited access to care. Unmeasured variables, including medication adherence, social support, and coping mechanisms, could also confound the observed associations.

Overall, our research shows that IBD severity has a close connection with the burden of mental health, and such variables as gender, age, disease activity, type, and family history are the determinants of psychological outcomes. These results indicate the relevance of psychosocial care in the management of IBD and contribute to the necessity of cultural interventions in Pakistan to support both physical and psychological health requirements in the population.

Study constraints

These findings should be interpreted in light of several limitations. First, the cross-sectional design does not allow for causal conclusions about the relationship between IBD severity and psychological burden; longitudinal studies would be needed to explore temporal and causal relationships. Second, convenience sampling from urban outpatient clinics may reduce generalizability, as rural populations, older adults, and patients with limited access to healthcare are underrepresented. Third, disease activity was assessed solely via participant self-report (mild/moderate/severe) without objective clinical, endoscopic, or laboratory validation, which may have introduced misclassification bias. Incorporating validated clinical indices or biomarkers (e.g., CRP, fecal calprotectin, Harvey-Bradshaw Index, or Mayo Score) in future studies would improve accuracy. Fourth, the study relied on self-reported questionnaires, which are subject to recall and response bias. Additionally, the HADS and SIBDQ were administered in English without formal linguistic or cultural validation, and although bilingual assistance was provided, comprehension and response accuracy may have been affected. Finally, potential confounding factors such as medication adherence, social support, and coping strategies were not evaluated, which could have provided a deeper understanding of the psychosocial determinants of mental health in IBD.

Future directions

Longitudinal designs should be employed in future studies to gain a deeper understanding of the directionality of the gut-brain relationship in IBD. Cultural aspects of stigma and coping may also be captured in qualitative studies of patients' lived experiences with stigma, supplementing quantitative results. Moreover, screening tools should be validated and translated into local languages to ensure greater accuracy and cultural acceptability. The usefulness of combined interventions, including cognitive behavioural therapy (CBT), stress management, or psychiatric support in addition to standard IBD management approaches, needs to be tested. Lastly, there may be a new mechanistic understanding of the association between genetic, microbiome, and inflammatory biomarkers and mental health outcomes.

## Conclusions

The current study demonstrates that anxiety and depression are highly prevalent among Pakistani patients with IBD, with disease severity, age, and gender being the most significant predictors of psychological burden. Females and younger adults were significantly associated with higher psychological burden, while higher IBD severity was strongly associated with worse quality of life and increased psychological distress. These findings underscore the importance of integrating mental health assessment into routine IBD care. Routine screening for anxiety and depression in IBD clinics could improve outcomes and enhance overall patient wellbeing.

## Appendices

Appendix 1

The questionnaire includes demographic and clinical data, such as age, sex, marital status (single or married), education level (less than university/beyond university), occupation, IBD type, duration (in years), comorbidities, and treatment modalities (Table 7).

**Table 7:** Demographic Questionnaire

Items	Responses
Age	-
Gender	-
Marital status	-
Education	-
Employment	-
Type of IBD	-
Duration since diagnosis	-
Current disease activity (self-reported)	-
Current treatment (select all that apply	-
History of IBD-related surgery	-
Family history of IBD	-
Any psychiatric treatment in past or present?	-

Appendix 2

Developed by Irvine et al. in 1996, the SIBDQ is a 10-item questionnaire that measures health-related quality of life (HRQol) in patients with inflammatory bowel disease (IBD) (Table 8)

**Table 8:** Short Inflammatory Bowel Disease (IBD) Questionnaire Developed by Irvine et al. (1996) [11].

Items	Responses
How often has the feeling of fatigue or being tired and worn out been a problem for you during the past 2 weeks?	-
How often during the last 2 weeks have you delayed or canceled a social engagement because of your bowel problem?	-
As a result of your bowel problems, how much difficulty did you experience doing leisure or sports activities during the past 2 weeks?	-
How often during the past 2 weeks have you been troubled by pain in the abdomen?	-
How often during the past 2 weeks have you felt depressed or discouraged?	-
Overall, in the past 2 weeks, how much of a problem have you had with passing large amounts of gas?	-
Overall, in the past 2 weeks, how much of a problem have you had maintaining or getting to the weight you would like to be?	-
How often during the past 2 weeks have you felt relaxed and free of tension?	-
How much of the time during the past 2 weeks have you been troubled by a feeling of having to go to the bathroom even though your bowels were empty?	-
How often during the past 2 weeks have you felt angry as a result of your bowel problem?	-

Appendix 3

Anxiety and depressive symptoms of IBD patients were evaluated using HADS (Table 9).

**Table 9:** Hospital Anxiety and Depression Scale (HADS) Developed by Zigmond and Snaith (1983) [12].

Items	Responses
I feel tense or “wound up.”	-
I still enjoy the things I used to enjoy.	-
I get a sort of frightened feeling as if something awful is about to happen.	-
I can laugh and see the funny side of things.	-
Worrying thoughts go through my mind.	-
I feel cheerful.	-
I can sit at ease and feel relaxed.	-
I feel as if I am slowed down.	-
I get a sort of frightened feeling like “butterflies” in the stomach.	-
I have lost interest in my appearance.	-
I feel restless as if I have to be on the move.	-
I look forward with enjoyment to things.	-
I get sudden feelings of panic.	-
I can enjoy a good book or radio or TV program.	-
